# Factors influencing Chinese public attitudes toward farm animal welfare

**DOI:** 10.3389/fpsyg.2023.1049530

**Published:** 2023-03-09

**Authors:** Bing Jiang, Wenjie Tang, Lihang Cui, Yanjiao Wei

**Affiliations:** ^1^College of Economics and Management, Northeast Agricultural University, Harbin, China; ^2^Development Research Center of Modern Agriculture, Northeast Agricultural University, Harbin, China; ^3^Business School, Yangzhou University, Yangzhou, China

**Keywords:** public attitudes, farm animal welfare, affective attitudes, cognitive attitudes, behavioral attitudes, demographic factors

## Abstract

A comprehensive understanding of current Chinese public attitudes toward farm animal welfare and the relevant influencing factors is essential for improving farm animal welfare and promoting further development of animal husbandry. The attitudes of 3,726 respondents in China were investigated using paper and online questionnaires. Three components (affective, cognitive, and behavioral) of attitudes toward farm animal welfare were assessed using 18 items designed based on the literature review. Influential factors of attitudes toward farm animal welfare were explored *via* tobit regression. The results revealed that the Chinese public not only considers farm animals to be emotional and sentient but are also sympathetic toward farm animals that suffer inhumane treatment. Although they have limited knowledge about farm animal welfare, the public believes improving farm animal welfare is beneficial, especially for food safety and human health. The Chinese public prefers regulation policies to incentive policies for improving farm animal welfare. The main factors influencing attitudes toward farm animal welfare included gender, age, education, monthly household income, area of residence, farm animal raising experience, and attention to farm animal welfare events. The effect of these influencing factors on attitudes varied. These findings provide a basis for improving Chinese public attitudes toward farm animal welfare. The implications of formulating and implementing effective policies to improve the Chinese public attitudes toward farm animal welfare were discussed.

## 1. Introduction

The recent COVID-19 pandemic has affected public attitudes toward the human–animal relationship and triggered Chinese reflection on animal welfare and protection (Chen et al., 2021; Platto et al., 2022). The impact of animal health on human health and the relationships between animal welfare and animal health are attracting increasing attention and generating more discussion across society (Gu et al., 2022). The call for the improvement of farm animal welfare has been growing stronger, largely due to its connection with food safety and quality (Carnovale et al., 2021). Numerous studies have highlighted that improved farm animal welfare and protection is not only an effective means to promote the development of China's modern animal husbandry but also important to ensure the safety and quality of animal products (Xiong and Wang, 2020; Cui et al., 2021).

Despite the benefits animal welfare provides, China has focused too much on economic development in past decades, and welfare concerns have often been neglected by the government (Wang and Gu, 2016). This fact is demonstrated by the absence of nationwide laws and official controls in the field of farm animal welfare (Sun et al., 2021). China is one of the largest producers and exporters of animal products globally, and the neglect of farm animal welfare has resulted in severe economic and social consequences, such as interval outbreaks of epizootic diseases, increasing problems with environmental pollution, a ban on animal product imports by developed countries, and recurring animal product safety and quality problems (You et al., 2014; Wang, 2018; Carnovale et al., 2021; Sun et al., 2021; Xiong et al., 2022). As the demand for animal products increases alongside the growth of the population, the impact of farm animal welfare on modern animal husbandry has gained increasing importance in recent years. Therefore, how to promote farm animal welfare in China has become an urgent issue to be solved.

According to the theory of planned behavior, attitude is the most proximal predictor of behavior in addition to social norms and perceived behavioral control (Ajzen and Fishbein, 1977). Applying this to the context of farm animal welfare, effective policies and legislation require the active expression and broad participation of the public. However, the improvement of farm animal welfare is also a process in which the government makes changes in response to public expectations and attitudes. As such, public attitudes toward farm animal welfare will influence farm animal welfare policies and legislation and play a role in determining the treatment of farm animals (Bertenshaw and Rowlinson, 2009; Hazel et al., 2011; Hemsworth et al., 2021). Public attitudes may be understood as the most potent driving force for farm animal welfare improvement. In Europe, for example, the increasing public concerns for farm animal welfare resulted in new, more stringent legal provisions from the European Union (Ostovic et al., 2017). Considering that the welfare of farm animals in China is still in its early stages, improving public attitudes toward farm animal welfare may be an important foundation for the development and implementation of policy and legislation (You et al., 2014; Platto et al., 2022).

Although the attitudes of the Chinese public toward farm animal welfare have received increasing attention in academia, there are still relatively few studies when compared to those regarding Europe, Oceania, and America, where farm animal welfare is well-developed (Sinclair et al., 2020). With urbanization, there has been a considerable social and spatial distance between the public and animal husbandry, and there is a lot of scope to improve Chinese attitudes toward farm animal welfare (You et al., 2014; Carnovale et al., 2021). Furthermore, a number of factors have been found to influence attitudes, which are dominated by demographic characteristics such as gender, age, education, income, area of residence, and occupation (You et al., 2014; Su and Martens, 2017; Carnovale et al., 2022). Previous studies on public attitudes toward farm animal welfare (whether in China or elsewhere) have considered attitude as a whole concept. These studies did not measure and compare public attitudes toward farm animal welfare from different dimensions. However, attitude is widely considered to be a multidimensional concept, consisting of affective attitude, behavioral attitude, and cognitive attitude (Rosenberg and Hovland, 1960; Eagly and Chaiken, 1993). Therefore, to accurately describe the Chinese public attitudes toward farm animal welfare, an appropriate multidimensional investigation and measurement of attitude are required (Ferrer-Urbina et al., 2021; Giménez-Espert et al., 2021).

In the current study, the Chinese public attitudes toward farm animal welfare were investigated and measured across the three dimensions, and the current status of the Chinese public attitudes toward farm animal welfare was analyzed and compared. Furthermore, the present study explored factors influencing the Chinese public attitudes toward farm animal welfare and analyzed the different effects of these factors on different dimensions of attitudes.

## 2. Theoretical background

### 2.1. Three-dimensional model of an attitude

Attitude is commonly defined as “an individual's overall evaluation of persons (including themselves), objects, and issues” (Petty and Wegener, 1998). Accordingly, “public attitudes toward farm animal welfare” can be defined as the overall evaluation of farm animal welfare among the public. The three-dimensional model of an attitude has suggested that attitude consists of an affective component, a behavioral component, and a cognitive component (Rosenberg and Hovland, 1960). The affective component of an attitude refers to the emotional evaluation of an object, and it reflects the emotional underpinnings of an attitude. The cognitive component of an attitude is defined as an individual's mental conceptualization of the object or their thoughts and beliefs about the object. The behavioral component of an attitude refers to the way of acting that allows making evaluations about the attitudinal object, such as intended behavior toward the attitude object (Zanna and Rempel, 1988; Eagly and Chaiken, 1993; Krischler and Pit-ten Cate, 2019; Ferrer-Urbina et al., 2021). In the current study, the three-dimensional model of an attitude was applied to assess the affective, cognitive, and behavioral components of the Chinese public attitudes toward farm animal welfare.

### 2.2. Literature review and hypotheses

#### 2.2.1. Chinese public attitudes toward farm animal welfare

In previous studies, the affective attitude of the Chinese public is ambiguous due to differences in survey time and place. Wang and Gu (2016) found that 39.7% of respondents believed raising large numbers of chickens in a small space is normal, and 51.0% of respondents believed bloodletting of live chickens is normal. They concluded that a considerable proportion of the Chinese public did not hold negative affective attitudes toward the inhumane treatment of farm animals. In contrast to this, Cui et al. (2021) found that the vast majority of the public hold negative affective attitudes toward scenes of inhumane treatment of dairy cattle that were reported in news. A survey conducted in 2011 also revealed that 69.7% of respondents thought that it is somewhat or extremely inappropriate to rear pigs on a cement floor, and 74.3% of respondents considered killing fowls near cages to be somewhat or extremely inappropriate (You et al., 2014).

It is generally understood that the Chinese public cognitive attitude toward farm animal welfare is negative. Wang and Gu (2016) found that more than 40.4% of respondents had never heard of the concept of farm animal welfare, and 44.9% of respondents had heard of farm animal welfare but did not understand the concept. Furthermore, Carnovale et al. (2021) found that nearly half of the respondents have never heard the term “animal welfare.”

All the previous studies have reached a consistent conclusion about the Chinese public behavioral attitude toward farm animal welfare. It has been reported that the public is generally willing to pay more for animal products with positive animal welfare attributes and that they have supported legislation on farm animal welfare (Wang and Gu, 2014; Chen et al., 2021; Cui et al., 2021).

#### 2.2.2. Influencing factors of public attitudes toward farm animal welfare

The influence of demographic characteristics on attitude has been explored in previous studies. (1) Gender: Heleski et al. (2004), Kupsala et al. (2015), and Wigham et al. (2020) found that women's attitudes toward farm animal welfare were more positive than men's. (2) Age: Kupsala et al. (2015) showed that young people expressed more positive attitudes toward farm animal welfare than older people. Randler et al. (2021) found that there was no difference in attitudes toward farm animal welfare between adolescents of different ages. (3) Education: You et al. (2014) revealed those with higher education had a greater understanding of farm animal welfare, and those with lower education were more indifferent to the inhumane treatment of farm animals. (4) Monthly household income: Ostovic et al. (2017) found that monthly household income was not a significant factor affecting the attitudes of veterinary students toward farm animal welfare, whereas You et al. (2014) found that Chinese citizens with higher monthly household income were more willing to support mandatory legislation on farm animal welfare and pay more for welfare-friendly animal products. (5) Area of residence: Spooner et al. (2014) found that there was no difference between the attitudes of residents in urban areas and rural areas toward farm animal welfare; however, Estevez-Moreno et al. (2021) found that urban consumers concerned more about farm animal welfare. (6) Occupation: It has been found that attitudes toward farm animal welfare differ significantly by occupation (Maria, 2006; You et al., 2014).

Additional factors, including animal-related experiences, dietary habits, and media exposure, have also been found to affect attitudes toward farm animal welfare. (1) Farm animal raising experience: Boogaard et al. (2006) and Kupsala et al. (2015) both found that the attitude toward farm animal welfare of individuals who have raised farm animals was more positive than that of those who have not. (2) Food consumption habit: Vegetarians were found to express more welfare concerns regarding farm animal practices (Ostovic et al., 2017; Ly et al., 2021). (3) Attention to farm animal welfare events or reports: Sinclair et al. (2018) found that negative media reports would induce negative emotions of the public, and Clark et al. (2016) also found that the attitudes of those who expressed more concern toward farm animal welfare were more positive.

In summary, several demographic characteristics, animal-related experiences, dietary habits, and media exposure should be accounted for as influencing factors of the Chinese public attitudes toward farm animal welfare. Based on the literature review earlier, the following hypotheses were proposed.

Hypothesis 1: Women's attitudes toward farm animal welfare are more positive than men's.

Hypothesis 2: Age has a significant negative influence on attitudes toward farm animal welfare.

Hypothesis 3: Education has a significant positive influence on attitudes toward farm animal welfare.

Hypothesis 4: Monthly household income has a significant positive influence on attitudes toward farm animal welfare.

Hypothesis 5: Those living in urban areas have more positive attitudes toward farm animal welfare than those living in rural areas.

Hypothesis 6: There are significant differences in attitudes toward farm animal welfare of those with different occupations.

Hypothesis 7: Experience raising farm animals has a significant positive influence on attitudes toward farm animal welfare.

Hypothesis 8: Food consumption habit has a significant positive influence on attitudes toward farm animal welfare.

Hypothesis 9: Attention to farm animal welfare events or reports has a significant positive influence on attitudes toward farm animal welfare.

## 3. Materials and methods

### 3.1. Questionnaire design

To measure attitudes toward farm animal welfare, a standardized questionnaire was developed. The questionnaire consisted of two parts. The first part of the questionnaire consisted of 10 items relating to influencing factors, including demographic characteristics, animal-related experiences, dietary habits, and media exposure. The second part of the questionnaire consisted of 18 items about attitudes toward farm animal welfare, which were designed based on the three-dimensional model of an attitude. All the items were adapted from well-established scales in previous studies, combined with the development of farm animal welfare in China. (1) Four items (AFF1–AFF4) were drawn from Wang and Gu (2016), Cui et al. (2021), and Platto et al. (2022) and were used to measure affective attitude; these included scenarios of inhumane treatment of farm animals and statements relating to animal sentience. (2) Six items (COG1–COG6) were drawn from You et al. (2014), Carnovale et al. (2021), and Estevez-Moreno et al. (2021) and were used to measure cognitive attitude; these included statements relating to the concept, importance, and effect of farm animal welfare. (3) Eight items (BEH1–BEH8) were drawn from Cui et al. (2021) and Estevez-Moreno et al. (2021) and were used to measure behavioral attitude; these were mainly statements relating to improving measures of farm animal welfare. A five-point Likert scale ranging from 1 = “strongly disagree” to 5 = “strongly agree” was used. High scores in all the dimensions correspond to a positive attitude. The items are described in Table 1.

**Table 1:** Measurement items and weight coefficients.

**Construct**	**Code**	**Items**	**Subjective weight**	**Objective weight**	**Comprehensive weight**
Affective attitude	AFF1	I believe it is cruel for workers to kick and beat dairy cows with iron pipes.	0.0475	0.0268	0.02243
	AFF2	I believe it is inhumane to raising a lot of chicken in a limited space.	0.0403	0.0251	0.01783
	AFF3	I believe farm animals feel pain in the same way that humans do.	0.0733	0.0313	0.04043
	AFF4	I believe farm animals are aware of their bodily sensation.	0.0749	0.0243	0.03208
Cognitive attitude	COG1	I believe I understand the concept of farm animal welfare.	0.0256	0.0282	0.01272
	COG2	I believe I understand the advantages of welfare-friendly animal products over ordinary animal products.	0.0364	0.0789	0.05061
	COG3	I believe farm animal welfare attributes of animal products are important.	0.0386	0.0511	0.03476
	COG4	I believe improving farm animal welfare has enormous ecological, economic, and social value.	0.0384	0.0364	0.02463
	COG5	I believe improving farm animal welfare is beneficial for food safety and quality.	0.0651	0.0572	0.06562
	COG6	I believe improving farm animal welfare is beneficial for human health.	0.0662	0.0292	0.03407
Behavioral attitude	BEH1	I believe it is necessary to legislate on farm animal welfare.	0.0671	0.0225	0.02661
	BEH2	I believe punishment are needed to prevent abuse in the treatment of farm animals.	0.0612	0.0546	0.05889
	BEH3	I believe professional and general education on farm animal welfare should be developed.	0.0529	0.0619	0.05771
	BEH4	I believe farm animal welfare training for farms and slaughters should be organized.	0.0623	0.0626	0.06873
	BEH5	I am willing to pay more for welfare-friendly animal products.	0.0686	0.1258	0.15209
	BEH6	I believe the government should subsidize farms and slaughters that improve farm animal welfare.	0.0655	0.0826	0.09535
	BEH7	I believe welfare-friendly animal products should be certified and labeled.	0.0522	0.1041	0.09576
	BEH8	I believe national standards for farm animal welfare is needed to be established.	0.0639	0.0974	0.10968

### 3.2. Data collection

The data used for this study were obtained from field surveys and online surveys conducted in July and August 2021. The field surveys were conducted by a group of 20 investigators who were recruited from the undergraduate and graduate students of the College of Economics and Management, Northeast Agricultural University. After unified training and post-training tests, these investigators returned to their hometowns to conduct face-to-face interviews using a structured questionnaire during the summer holiday. The samples were collected from public places including shops, supermarkets, parks, and squares. The selection of respondents followed the principle of random sampling. The online surveys were conducted through a professional web-based questionnaire platform. The questionnaire was distributed using a snowball sampling strategy through WeChat, a popular messaging app. Participants were informed that participation was voluntary, refusal to participate would have no effect on them, the survey would not collect personal contacts or identifying information, and the data would be kept strictly confidential and would only be used for research purposes.

A total of 4,000 questionnaires were distributed, comprising 3,000 paper questionnaires and 1,000 online questionnaires, and missing data or poor-quality questionnaires were eliminated. Ultimately, 3,726 valid questionnaires were collected, with a valid response rate of 93.2%. The final proportion of paper and online questionnaires was approximately 3:1, with 2,795 paper questionnaires and 931 online questionnaires. One hundred and three rural and urban areas under the jurisdiction of 31 provinces of China (excluding Hong Kong, Macao, and Taiwan) were covered in the investigation.

### 3.3. Statistical analyses

The statistical analysis involved four steps: (1) Descriptive statistical analyses were performed; (2) the reliability of the scale was examined using Cronbach's alpha coefficients, and the construct validity of the scale was examined using Kaiser–Meyer–Olkin (KMO) values and Bartlett's test of sphericity; (3) mean scores for each item and attitude construct of the scale were calculated to analyze public overall attitudes, affective attitudes, cognitive attitudes, and behavioral attitudes toward farm animal welfare; (4) tobit models were designed to identify factors influencing public overall attitudes, affective attitudes, cognitive attitudes, and behavioral attitudes toward farm animal welfare. An analytic hierarchy process (AHP) and an entropy method were used to determine the weight of each item before regression analysis, and thus, the scores of attitudes were calculated as explained variables (Peng, 2022; Shen and Liao, 2022). After normalizing the scores, collinearity was tested using variance inflation factors (VIFs). The aforementioned steps were performed by STATA software version 15 and SPSS software version 24.

### 3.4. Model design

The specific form of the tobit model is given as follows:


$$\begin{array}{l} Y^{*}=\alpha +\sum_{j=1}^{n}\beta_{j}X_{j}+\varepsilon \\ \;Y=\{\begin{array}{cc} Y^{*}, & if\;Y^{*}>0\\ 0,\; & if\;Y^{*}\leq 0 \end{array}, \end{array}$$

where Y^*^ is the score of public attitudes toward farm animal welfare, α is a constant term, β_*j*_ is the coefficients to be estimated, and *j* = 1, 2, …, *n*, *X*_*j*_ are factors influencing public attitudes toward farm animal welfare, ε represents the random error term where ε~*N*(0, σ^2^), and *Y* is the observed variable of Y^*^.

Explained variables are overall attitude score, affective attitude score, cognitive attitude score, and behavioral attitude score. The scores were calculated using the scores and weights of each item on the scale. The weight coefficients determined by the AHP and the entropy method are shown in Table 1. The detailed procedures of weighting are available in Supplementary material. For ease of regression analysis, the scores were normalized.

Explanatory variables are influencing factors, including demographic characteristics, animal-related experiences, dietary habits, and media exposure. (1) Demographic characteristics included gender, age, education, monthly household income, area of residence, and occupation. (2) Referring to Kupsala et al. (2015), animal-related experiences were measured based on farm animal raising experiences. (3) Referring to Zhang et al. (2021), dietary habits were measured by food consumption habits. (4) Referring to Wang and Gu (2014), media exposure was measured by attention to farm animal welfare events or reports. Considering that public attitudes toward farm animal welfare may be affected by macro-level factors in different regions of residence, such as socioeconomic development or cultural practices, region-fixed effects were thus controlled. Definitions and assignments of variables are shown in Table 2.

**Table 2:** Variable definitions and assignments.

**Variable classification**	**Variable definitions and assignments**
Explained variable	Overall attitude score
	Affective attitude score
	Cognitive attitude score
	Behavioral attitude score
Explanatory variables	Gender: men = 1, women = 0
	Age: 18–20 = 1, 21–30 = 2, 31–40 = 3, 41–50 = 4, 51–60 = 5, 61–80 = 6
	Education: primary school and below = 1, junior middle school = 2, high school = 3, college = 4, undergraduate = 5, postgraduate = 6
	Monthly household income (in RMB): < 4,001 = 1, 4,001–8,000 = 2, 8,001–12,000 = 3, 12,001–16,000 = 4, >16,000 = 5
	Area of residence: urban area = 1, rural area = 0
	Occupation: unemployed = 1, students = 2, farmers = 3, self-employ = 4, enterprise staff = 5, public institution = 6, civil servant = 7, retired = 8
	Farm animal raising experience: yes = 1, no = 0
	Food consumption habit: plant-based dietary = 1, balanced dietary = 2, animal-based dietary = 3
	Attention to farm animal welfare events or reports: never = 1, occasionally = 2, sometimes = 3, often = 4, always = 5

## 4. Results

### 4.1. Sample characteristics

The ratio of men to women was 1.04:1. More than half of the respondents (*n* = 2,164, 58.1%) were aged between 18 and 40 years. The education of the respondents was generally college-level and above (*n* = 2,114, 56.7%). Approximately two-thirds (*n* = 2,484, 66.7%) of the respondents reported monthly household income ranging from RMB¥ 4,001 to 16,000 (approximately ranging USD$ 571.6 to 2,285.7 at the exchange rate of RMB¥ 7 to USD$ 1). The majority of the respondents (*n* = 2,419, 64.9%) were living in urban areas. Self-employed respondents made up the majority of the sample (*n* = 828, 22.2%), followed by students (*n* = 770, 20.7%). The respondents were from 31 provinces of seven regions. The specific location sources and the number of samples were as follows: 468 respondents were from North China (114 from Beijing, 68 from Tianjin, 106 from Hebei, 73 from Shanxi, and 107 from Inner Mongolia), 813 respondents were from Northeast China (198 from Liaoning, 154 from Jilin, and 461 from Heilongjiang), 537 respondents were from East China (30 from Shanghai, 107 from Jiangsu, 82 from Zhejiang, 78 from Anhui, 53 from Fujian, 59 from Jiangxi, and 128 from Shandong), 546 respondents were from Central China (243 from Henan, 142 from Hubei, and 161 from Hunan), 502 respondents were from South China (339 from Guangdong, 136 from Guangxi, and 27 from Hainan), 538 respondents were from Southwest China (84 from Chongqing, 219 from Sichuan, 102 from Guizhou, 123 from Yunnan, and 10 of Xizang), and 322 respondents were from Northwest China (123 from Shaanxi, 77 from Gansu, 19 from Qinghai, 23 from Ningxia, and 80 from Xinjiang). Respondents living in Northeast China comprised the majority of the sample (21.8%), and those living in the Northwest China were the least represented (8.6%).

The gender and area of residents approximated the seventh National Census in 2020; however, the current sample was younger and more educated and had higher monthly household income. Based on previous studies (You et al., 2014; Wang and Gu, 2016; Cui et al., 2021), individuals who are younger, are more educated, and have higher monthly household income are the primary target audience of farm animal welfare, which is consistent with the theory of innovation diffusion (Rogers, 1962). Therefore, the sample was somewhat representative. Sample characteristics are presented in Table 3.

**Table 3:** Sample characteristics.

**Characteristics**		**No. of residents**	**% of sample**
Gender	Men	1,900	51.0
	Women	1,826	49.0
Age	18–20	288	7.7
	21–30	1,121	30.1
	31–40	755	20.3
	41–50	857	23.0
	51–60	401	10.8
	61–80	304	8.2
Education	Primary school and below	512	13.7
	Junior middle school	556	14.9
	High school	544	14.6
	College	734	19.7
	Undergraduate	1,048	28.1
	Postgraduate	332	8.9
Monthly household income	<4,001 RMB	745	20.0
	(<571.6 USD)		
	4,001–8,000 RMB	785	21.1
	(571.6–1,142.9 USD)		
	8,001–12,000 RMB	932	25.0
	(1,143.0–1,714.3 USD)		
	12,001–16,000 RMB	767	20.6
	(1,714.4–2,285.7 USD)		
	>16,000 RMB	497	13.3
	(>2,285.7 USD)		
Area of residence	Urban area	2,419	64.9
	Rural area	1,307	35.1
Occupation	Unemployed	105	2.8
	Students	770	20.7
	Farmers	526	14.1
	Self-employed	828	22.2
	Enterprise staff	600	16.1
	Public institution	326	8.7
	Civil servant	258	6.9
	Retired	313	8.4
Region of residence	North China	468	12.6
	Northeast China	813	21.8
	East China	537	14.4
	Central China	546	14.7
	South China	502	13.5
	Southwest China	538	14.4
	Northwest China	322	8.6

The monthly household income is converted from RMB into USD at the exchange rate of 7 RMB to 1 USD.

### 4.2. Reliability and validity analysis

The reliability of the scale was assessed by Cronbach's alpha. The results showed that Cronbach's alphas of affective attitude items, behavioral attitude items, and cognitive attitude items were 0.718, 0.787, and 0.768, respectively, and the overall Cronbach's alpha was 0.867. Cronbach's alphas obtained were all above 0.7, which indicates that the reliability of items is considered satisfactory. The KMO measure of sampling adequacy and Bartlett's test of sphericity were used to assess the construct validity of the scale. The results showed that the KMO-value (0.867) is >0.8 and the results of Bartlett's test of sphericity ensured a highly significant *p*-value (*p* < 0.001), which indicates that the construct validity of items is considered satisfactory. All scales passed the tests of reliability and validity.

### 4.3. Chinese public attitudes toward farm animal welfare

#### 4.3.1. Overall attitude

Descriptive statistics for scale scores are reported in Table 4. The mean scale score of the respondents' attitudes toward farm animal welfare was 3.3. Respondents scored highly on the affective component of an attitude, with the mean scale score of affective attitudes being 4.1. The score of the behavioral component of an attitude was slightly lower than affective attitudes, at 3.5. The cognitive component of an attitude had the lowest score of the three components, with the mean scale score being 2.5.

**Table 4:** Descriptive statistics for scale scores.

**Items**	**Strongly disagree**	**Disagree**	**Neutral**	**Agree**	**Strongly agree**	**Mean**
AFF1	113	159	514	1,223	1,717	4.1
AFF2	103	148	612	1,142	1,721	4.1
AFF3	239	252	719	700	1,816	4.0
AFF4	180	175	778	850	1,743	4.0
**Affective attitude**						4.1
COG1	2,603	438	358	234	93	1.6
COG2	2,667	432	372	153	102	1.5
COG3	2,375	572	318	264	197	1.7
COG4	1,582	490	231	641	782	2.6
COG5	277	300	481	2,181	487	3.6
COG6	417	468	516	1,986	339	3.4
**Cognitive attitude**						2.4
BEH1	296	368	582	1,451	1,029	3.7
BEH2	264	339	484	945	1,694	3.9
BEH3	424	513	610	1,003	1,176	3.5
BEH4	598	612	409	1,112	995	3.3
BEH5	221	495	771	1,158	1,081	3.6
BEH6	618	415	1,218	614	861	3.2
BEH7	367	510	843	1,050	956	3.5
BEH8	282	378	991	1,061	1,014	3.6
**Behavioral attitude**						3.5
**Overall attitude**						3.3

#### 4.3.2. Affective component of an attitude

As shown in Table 4, most of the respondents believed it is cruel for workers to kick and beat dairy cows with iron pipes, with 78.9% of the respondents (*n* = 2,940) strongly agreeing (*n* = 1,717, 46.1%), or agreeing (*n* = 1,223, 32.8%). Approximately three-quarters (*n* = 2,863, 76.8%) of the respondents either strongly agreed (*n* = 1,721, 46.2%) or agreed (*n* = 1,142, 30.6%) that it is inhumane to raise a lot of chickens in a limited space. Among respondents, 48.7% strongly agreed (*n* = 1,816) that farm animals feel pain in the same way that humans do, and 18.8% expressed some level of agreement (*n* = 700). Nearly half of the respondents (*n* = 1,743, 46.8%) strongly agreed with the statement that farm animals are aware of their bodily sensations, and more than a fifth (*n* = 850, 22.8%) were in agreement with this statement.

#### 4.3.3. Cognitive component of an attitude

As presented in Table 4, a large majority of the respondents (*n* = 3,041, 81.6%) did not understand the concept of farm animal welfare. Similarly, the vast majority of the respondents (*n* = 3,099, 83.2%) did not understand the advantages of welfare-friendly animal products over ordinary animal products. Only 12.4% of the respondents (*n* = 461) either strongly agreed (*n* = 197, 5.3%) or agreed (*n* = 264, 7.1%) with the perspective that farm animal welfare attributes of animal products are important. Regarding the beneficial effects of improving farm animal welfare on ecology, economy, and society, 38.2% of the respondents (*n* = 1,423) strongly agreed (*n* = 782, 21.0%) or agreed (*n* = 641, 17.2%) with the statement. In contrast, more respondents strongly agreed or agreed with the perspective that improving farm animal welfare is beneficial for food safety, quality (*n* = 2,668, 71.6%), and human health (*n* = 2,325, 62.4%).

#### 4.3.4. Behavioral component of an attitude

As displayed in Table 4, 66.6% of the respondents (*n* = 2,480) strongly agreed (*n* = 1,029, 27.6%) or agreed (*n* = 1,451, 38.9%) with the opinion that it is necessary to legislate on farm animal welfare. Almost half of the respondents (*n* = 1,694, 45.5%) strongly agreed that punishment is needed to prevent abuse in the treatment of farm animals, with 25.4% agreeing (*n* = 945). More than half of the respondents (*n* = 2,179, 58.5%) strongly agreed (*n* = 1,176, 31.6%) or agreed (*n* = 1,003, 26.9%) that professional and general education on farm animal welfare should be developed. Similarly, 56.5% of the respondents (*n* = 2,107) strongly agreed (*n* = 995, 26.7%) or agreed (*n* = 1,112, 29.8%) that farm animal welfare training for farms and slaughters should be organized. A small proportion of the respondents (*n* = 716, 19.2%) were not willing to pay more for welfare-friendly animal products, while most respondents (*n* = 2,239, 60.1%) were willing to pay. When asked whether the government should subsidize farms and slaughters that improve farm animal welfare, a minority of the respondents (*n* = 1,033, 27.7%) strongly disagreed (*n* = 618, 16.6%) or disagreed (*n* = 415, 11.1%) with this proposal, with more respondents (*n* = 1,475, 39.6%) strong agreeing (*n* = 861, 23.1%) or agreeing (*n* = 614, 16.5%). With regard to certifying and labeling welfare-friendly animal products, 25.7% of the respondents (*n* = 956) were in strong agreement with this statement and 28.2% were in agreement (*n* = 1,050). Over half of the respondents (*n* = 2,075, 55.7%) either strongly agreed (*n* = 1,014, 27.2%) or agreed (*n* = 1,061, 28.5%) with the statement that national standards for farm animal welfare need to be established.

### 4.4. Influencing factors of Chinese public attitudes toward farm animal welfare

Table 5 presents the estimated results of Model 1–Model 4, whose explained variables were overall attitude score (Model 1), affective attitude score (Model 2), cognitive attitude score (Model 3), and behavioral attitude score (Model 4). The result of the collinearity test shows that the maximum VIF is 1.92, and the mean VIF is far <5, indicating that there is no collinearity among the explanatory variables. The result of the goodness-of-fit test shows that all models fit reasonably according to the significance of the LR chi-square.

**Table 5:** Estimated results of influencing factors of public attitudes toward farm animal welfare.

**Influencing factors**	**Model 1**	**Model 2**	**Model 3**	**Model 4**
	**Overall attitude**	**Affective attitude**	**Cognitive attitude**	**Behavioral attitude**
Gender	−0.0967^***^	−0.0965^***^	−0.0110	−0.0434
	(0.0228)	(0.0201)	(0.0276)	(0.0271)
Age	−0.0630^***^	−0.0546^***^	−0.0135	−0.0232
	(0.0167)	(0.0147)	(0.0203)	(0.0199)
Education	0.0576^***^	0.0157	0.0602^***^	0.0546^***^
	(0.0139)	(0.0122)	(0.0169)	(0.0166)
Monthly household income	0.0091	−0.0264^**^	0.0057	−0.0343^*^
	(0.0127)	(0.0111)	(0.0153)	(0.0150)
Area of residence	0.0709^***^	0.0466^***^	0.0251	0.0511^**^
	(0.0152)	(0.0133)	(0.0183)	(0.0181)
Occupation	0.0102	0.0047	0.0213	0.0152
	(0.0118)	(0.0103)	(0.0142)	(0.0140)
Farm animal raising experience	0.0192	−0.0206	0.0134	−0.0431^*^
	(0.0157)	(0.0137)	(0.0191)	(0.0187)
Food consumption habit	−0.0155	0.0067	0.0079	0.0181
	(0.0170)	(0.0133)	(0.0184)	(0.0181)
Attention to farm animal welfare events or reports	0.0196	0.0280^**^	0.0084	0.0474^***^
	(0.0120)	(0.0105)	(0.0145)	(0.0142)
Region fixed effects	Control	Control	Control	Control
Constant	0.4441^***^	0.5007^***^	0.4843^***^	0.4769^***^
	(0.1072)	(0.0948)	(0.1301)	(0.1272)
Sample size	3,726	3,726	3,726	3,726
Log likelihood	2,894.67	2,804.36	2,348.28	2,650.61
LR chi^2^	1,161.89^***^	1,153.28^***^	941.61^***^	1,105.25^***^
Prob > chi^2^	0.0000	0.0000	0.0000	0.0000
Pseudo *R*^2^	0.2897	0.2594	0.1510	0.2361

* *p* < 0.05,

** *p* < 0.01, and

*** *p* < 0.001.

Standard error is in parentheses.

#### 4.4.1. Influencing factors of the overall attitude

In Model 1, gender had a significant negative effect on overall attitude (β = −0.0967, *t* = −4.2412, *p* < 0.001), indicating that female respondents had more positive attitudes toward farm animal welfare than male respondents. Age had a significant negative effect on overall attitude (β = −0.0630, *t* = −3.7725, *p* < 0.001), with younger age being associated with more positive attitudes toward farm animal welfare. There was a significant positive effect of education on overall attitude, indicating that better education had a direct association with more positive attitudes toward farm animal welfare (β = 0.0576, *t* = 4.1439, *p* < 0.001). The interaction between the area of residence and overall attitudes was significant, indicating that respondents living in urban areas tend to have more positive attitudes toward farm animal welfare (β = 0.0709, *t* = 4.6645, *p* < 0.001). The effects of monthly household income, occupation, farm animal raising experience, food consumption habit, and attention to farm animal welfare events or reports on overall attitude were not significant.

#### 4.4.2. Influencing factors of affective attitude

Similar to Model 1, gender (β = −0.0965, *t* = −4.8010, *p* < 0.001) and age (β = −0.0546, *t* = −3.7143, *p* < 0.001) were significantly and negatively associated with affective attitude, and area of residence (β = 0.0466, *t* = 3.5038, *p* < 0.001) had a significant positive impact on affective attitude. In Model 2, a significant negative relationship between monthly household income and affective attitude was found (β = −0.0264, *t* = −2.3784, *p* < 0.01), indicating that respondents with a lower monthly household income had more positive affective attitudes. Attention to farm animal welfare events or reports had a significant positive impact on affective attitude (β = 0.0280, *t* = 2.6667, *p* < 0.01), indicating that the greater attention paid to farm animal welfare events or reports, the more positive the affective attitude toward farm animal welfare. No significant effects of education, occupation, farm animal raising experience, and food consumption habit on affective attitude were observed.

#### 4.4.3. Influencing factors of cognitive attitude

In Model 3, education was the only significant factor influencing cognitive attitude. Education had a significant positive effect on cognitive attitude (β = 0.0602, *t* = 3.5621, *p* < 0.001), which indicated that respondents with higher educational attainments had more positive cognitive attitudes toward farm animal welfare. No significant effects were found for the other factors.

#### 4.4.4. Influencing factors of behavioral attitude

Similar to Model 1, education (β = 0.0546, *t* = 3.2892, *p* < 0.001) and area of residence (β = 0.0511, *t* = 2.8232, *p* < 0.01) had significant positive effects on behavioral attitude in Model 4. A significant negative influence of monthly household income on behavioral attitude was observed (β = −0.0343, *t* = −2.2867, *p* < 0.05), indicating that respondents with a lower monthly household income had more positive behavioral attitudes. Farm animal raising experience had a significantly negative impact on behavioral attitude (β = −0.0431, *t* = 2.3048, *p* < 0.05), indicating that respondents who have raised farm animals have more negative behavioral attitudes. Attention to farm animal welfare events or reports had a significant positive impact on behavioral attitude (β = 0.0474, *t* = 3.3380, *p* < 0.001), indicating that the greater the attention paid to farm animal welfare events or reports, the more positive the behavioral attitude toward farm animal welfare. There was no significant effect of gender, age, occupation, or food consumption habits on behavioral attitude.

## 5. Discussion

Of the three dimensions of an attitude, the Chinese public affective attitudes toward farm animal welfare are the most positive. From the aforementioned results, the Chinese public not only consider farm animals to be emotional and sentient but are also sympathetic toward farm animals suffering from inhumane treatment. The public's affective attitudes toward farm animal welfare originate from the human ability to empathize with animals (Daly and Morton, 2018). This is the ability to understand and feel animal situations and their psychology, which is a universal and innate ability of human beings (Yang and Dong, 2022).

Regarding cognitive attitude, the results show that, although the Chinese public has limited knowledge about farm animal welfare, they believe improving farm animal welfare is beneficial, especially for food safety and human health. This is consistent with the conclusion of Carnovale et al. (2021). In other words, the Chinese public cares about the wellbeing of farm animals and its positive effect on and importance for food quality and safety (Platto et al., 2022). Farm animal welfare is a relatively new concept in China, and information regarding farm animal welfare is very limited (Lu et al., 2013; You et al., 2014). The concept has been met with varying degrees of doubt, resistance, or ridicule, especially when the term “animal welfare” comes up, for example, by confusing the concept of animal rights with human rights (Cao, 2020; Miao et al., 2021). In addition, farm animal welfare attributes have been considered less important. This may be because, when it comes to purchasing animal products, the public are more focused on freshness and taste rather than the welfare of the animal (Han and Zhang, 2015).

Regarding behavioral attitude, the results reflected to some extent the Chinese public preferences for interventions or policies for improving farm animal welfare. Farm animal welfare is not only a moral issue but also a legal issue (Verrinder et al., 2019). The Chinese public support legislation for farm animal welfare and punishment to prevent abuse in the treatment of farm animals. These attitudes have been confirmed in other studies (You et al., 2014; Carnovale et al., 2021). As a quasi-public good, a significant expense is needed to improve farm animal welfare (Fernandes et al., 2021). However, a small proportion of the public is reluctant to pay the potential cost of farm animal welfare improvement, which may include paying premiums or taxes. Similar results were obtained by Lai et al. (2018) and Xu et al. (2019). A comprehensive farm animal welfare education system has not yet been formed in China, and only some agricultural colleges and universities offer farm animal welfare courses. Some large enterprises in animal husbandry have begun farm animal welfare training for major bodies in the supply chain and now require the disclosure of related information (Sinclair et al., 2019), but the Chinese public does not have strong opinions about this, and approximately a third of them are ambivalent to or disagree with the practice. Welfare-friendly animal products are credence goods, and the Chinese public believes welfare-friendly animal products should be certified and labeled to eliminate information asymmetry. National standards for farm animal welfare must be established to regulate the market. This finding is consistent with Xu et al. (2022). In summary, it is clear that the Chinese public prefers regulation policies to incentive policies for improving farm animal welfare.

The overall attitudes and affective attitudes of women were found to be more positive than men. This is consistent with Heleski et al. (2004), Kupsala et al. (2015), and Wigham et al. (2020). Generally, women are more emotional than men and are more likely to generate emotional associations and emotional expressions; hence, the female public expresses more empathy for farm animals (Lutz, 2016; Mazas and Fernandez-Manzanal, 2019). Meanwhile, women tend to be the main buyers of household food and are more sensitive and concerned with the welfare of farm animals and its impact on the quality and safety of animal products (Estevez-Moreno et al., 2021). Thus, Hypothesis 1 is verified.

The overall attitudes and affective attitudes of the younger public were found to be more positive than the older public, supporting Hypothesis 2. Farm animal welfare is a relatively novel concept in China (You et al., 2014). Compared with the elderly, young people were more willing to try, accept, and favor new ideas. This is consistent with Wang and Gu (2016) and Cui et al. (2021).

The overall attitudes, cognitive attitudes, and behavioral attitudes of the more educated public were found to be more positive than those with less education, consistent with findings from You et al. (2014). More educated people usually have broader knowledge and a deeper understanding of issues related to animal husbandry development and farm animal welfare (Rucinque et al., 2017). Therefore, they have more information and knowledge about how to promote the development of animal husbandry and improve farm animal welfare. In addition, the more educated people were found to be more aware of animal protection responsibility; they understand the importance of and long-term benefits of improving farm animal welfare to themselves and society (Clark et al., 2017). These results verify Hypothesis 3.

Affective attitudes and behavioral attitudes of the public with a lower monthly household income were found to be less positive than those with higher incomes. Monthly household income usually represents the economic status of the public. According to Kendall et al. (2006), economically disadvantaged groups tend to regard farm animals as disadvantaged groups. They tend to show more care and sympathy for farm animals and hope to take a series of measures to improve farm animal welfare. This conclusion is supported by findings from Phillips et al. (2012) and Boaitey and Minegishi (2020), and support is thus found for Hypothesis 4.

Overall attitudes, affective attitudes, and behavioral attitudes of the public living in urban areas were found to be more positive than those living in rural areas, which is consistent with findings from Ostovic et al. (2017). Animal husbandry is located mostly in rural areas, and those living in urban areas may not understand the real welfare status of farm animals, due to the social and spatial distance between themselves and animal husbandry (Boogaard et al., 2011). In addition, the lifestyles of modern technological and industrialized societies lead those living in urban areas to be more eager to live in harmony with nature; therefore, they become more concerned and sympathetic toward farm animals and hope to improve farm animal welfare (Estevez-Moreno et al., 2021). Meanwhile, animal husbandry may be a source of income for those living in rural areas, and as such, they may express more interest in the profitability of farm animals rather than in their welfare. Hence, Hypothesis 5 is also accepted.

The coefficient of occupation was not statistically significant; however, the mean of students' overall attitude scale scores was the highest, and farmers' overall attitude scale scores were the lowest. According to the theory of innovation diffusion (Rogers, 1962), students generally have the characteristics of younger age, higher education, less economic pressure, diversified value orientation, active thinking, and strong innovation ability, and they comprise a large proportion of proponents of farm animal welfare. In the context of the current shared rural culture, farmers have mostly viewed farm animals as a natural resource that can be exploited, rather than protected objects, regardless of whether animal husbandry is their main livelihood (Fan and Hong, 2015). Overall, these results support Hypothesis 6.

Behavioral attitudes of members of the public who have raised farm animals were found to be less positive. This finding is contrary to Boogaard et al. (2006) and Kupsala et al. (2015). Experience with pets might promote positive attitudes toward animals and negative attitudes toward their use for human consumption (Menor-Campos et al., 2019). However, different from pets, farm animal raising experience is always associated with the public who have relied heavily on raising farm animals as their primary livelihood activity (Spooner et al., 2014). These individuals usually have a more intuitive and deeper understanding of the welfare of farm animals, and they have a more objective judgment on the cost and difficulty of improving farm animal welfare. Therefore, their willingness to improve farm animal welfare is weaker (Wang and Gu, 2016; Cui et al., 2021). Therefore, Hypothesis 7 is accepted.

The coefficient of food consumption habit was not statistically significant. Vegetarians tend to match their eating habits to their ethics and are in support of animal rights as well as human rights (Miao et al., 2021). In the current study, however, there were nearly no differences in the attitudes toward farm animal welfare between members of the public with different food consumption habits. This might be because the proportion of vegetarians in the respondents was too small to yield any meaningful results. There may also be variation among the ethical ideologies of vegetarians, with some having a greater tolerance of farm animal suffering (Su and Martens, 2017). Therefore, Hypothesis 8 is rejected.

Cognitive attitudes and behavioral attitudes of the public who focus on events or reports related to farm animal welfare were found to be more positive. In the Chinese media, farm animal welfare events are mostly animal abuse events, and farm animal welfare reports focus mostly on the negative effects of ignoring farm animal welfare (Lu et al., 2013; Mu and Zhou, 2021). Therefore, the public who pays attention to these reports obtain more negative information, and they express more concern and compassion for farm animals and a willingness to improve farm animal welfare. This finding is consistent with the findings of Clark et al. (2016) and Sinclair et al. (2018). Thus, Hypothesis 9 is accepted.

## 6. Conclusion and implications

The study draws the following conclusions: (1) The Chinese public attitudes toward farm animal welfare consists of an affective component, cognitive component, and behavioral component. Their affective attitudes toward farm animal welfare are more positive than behavioral attitudes and their cognitive attitudes are the least positive. (2) The Chinese public not only considers farm animals to be emotional and sentient but is also sympathetic toward farm animals suffering from inhumane treatment. Although the Chinese public has limited knowledge about farm animal welfare, they believe that improving farm animal welfare is beneficial, especially for food safety and human health. The Chinese public prefers regulation policies to incentive policies for improving farm animal welfare. (3) Gender, age, education, and area of residence are significant factors influencing overall attitude; gender, age, monthly household income, area of residence, and attention to farm animal welfare events have a significant impact on affective attitude; education was the only significant factor influencing cognitive attitude; education, monthly household income, area of residence, farm animal raising experience, and attention to farm animal welfare events significantly influence behavioral attitude. Of these, the impacts of gender, age, monthly household income, and farm animal raising experience on attitudes toward farm animal welfare are negative, and the effects of education, area of residence, and attention to farm animal welfare events are positive.

Animal welfare is a responsibility that must be shared between governments, communities, the people who own, care for, and use animals, civil society, educational institutions, veterinarians, and scientists. Mutual recognition and constructive engagement among parties are necessary to achieve sustained improvements in animal welfare (World Organisation for Animal Health., 2017). Therefore, based on the earlier research conclusions, the current study suggests several steps to improve public attitudes toward farm animal welfare:

(1) Creating a social atmosphere of concern for farm animal welfare to foster public affective attitudes.

The government should continue to promote media publicity, through a long-term, sustained effort to spread information about farm animal welfare in news reports, thematic interviews, and public service advertisements. This information should include knowledge about the positive treatment of animals and negative portrayals of the inhumane treatment of farm animals. In news media, improving public opinions about farm animal welfare can be undertaken in the form of information push, topic discussion, and video clips that the public pays attention to and participates in.

In addition to government initiatives, the agricultural and livestock sectors should jointly take charge of undertaking publicity activities. The main purposes of these activities are the following: First, cultivating the consumption of welfare-friendly animal products with a target audience of the public who are women, are more educated, have higher monthly household income, and are living in urban areas. Second, shaping the awareness of farm animal welfare protection in the primary audience, that is, members of the public who are more educated and living in urban areas. Third, improving the animal welfare literacy of those who are women, are younger, have higher monthly household income, live in urban areas, and pay more attention to farm animal welfare events or reports.

(2) Strengthening farm animal welfare education to improve public cognitive attitudes.

First, the education sector should encourage conducting farm animal welfare school education. Farm animal welfare courses should be created in various professional courses in Agricultural and Forestry universities and research institutes. This would enable students and researchers to receive theoretical teaching and practical training about farm animal welfare.

Second, the agricultural and livestock sectors should encourage enterprises and institutions to conduct farm animal welfare education. Skills coaching and training should be offered to ensure all practitioners have up-to-date knowledge and awareness of farm animal welfare. Meanwhile, it is necessary to introduce qualification appraisal systems for farm animal welfare knowledge and skills during the hiring and performance evaluation processes.

Third, farm animal welfare science should be popularized. In terms of online science popularization, interesting and accessible scientific information about farm animal welfare should be continuously released on online platforms and social media, such as short videos, live videos, and blogs. Its content should cover knowledge about farm animal welfare, typical cases, and affecting events of inhumane treatment of farm animals, as well as expert interpretations and policy advocacy. In terms of offline science popularization, “Open Days on Farm Animal Welfare” could be set up in museums, zoos, and sightseeing pastures. Public lectures could be set up in parks, squares, supermarkets, and other crowded places. Meanwhile, scientific knowledge and methods to improve farm animal welfare can be disseminated by setting up exhibition boards, distributing manuals, and playing videos. These activities will require multiagent collaboration, especially the participation of enterprises. The sustainability of these activities could be ensured while enterprises are advertising.

(3) Introducing and implementing farm animal welfare policies to cater to public behavioral attitudes.

The government should introduce incentive policies for improving farm animal welfare. Reasonable and appropriate compensation strategies should be established according to actual costs and profits of farm animal welfare improvement. The incentives can take multiple forms, including direct subsidies, subsidies relating to equipment purchasing, tax deductions, and participation in government programs.

Furthermore, the government should introduce regulation policies for improving farm animal welfare. The cooperation of the agricultural and livestock sectors, research institutes, universities, industry associations, and enterprises is required to establish farm animal welfare standards. Such standards should be in accordance with the actual development of Chinese animal husbandry, and the standards of international organizations and developed countries must also be considered as a reference. In addition, it could be useful to establish a certified label implemented by third-party agencies. Those producing welfare-friendly animal products that meet the standards can voluntarily apply for certification.

## Data availability statement

The original contributions presented in the study are included in the article/Supplementary material, further inquiries can be directed to the corresponding author/s.

## Ethics statement

The studies involving human participants were reviewed and approved by Northeast Agricultural University. The patients/participants provided their written informed consent to participate in this study.

## Author contributions

BJ: conceptualization, methodology, resource, data curation, supervision, writing—original draft, and writing—reviewing and editing. WT and LC: investigation and writing—reviewing and editing. YW: writing—reviewing and editing. All authors contributed to the article and approved the submitted version.
