# Supplementary material for: Factors influencing Chinese public attitudes toward farm animal welfare
Source: Front Psychol. 2023 Mar 9;14:1049530. doi: 10.3389/fpsyg.2023.1049530 (PMC10035598; doi:10.3389/fpsyg.2023.1049530)
Supplement: Supplementary file 1 [file Data_Sheet_1.docx]

Supplementary Material

# Procedure of weighting

The AHP is used to obtain subjective weight according to expert opinions, and its evaluation results are logical and credible. The entropy method is used to obtain the objective weight according to the data itself, and its evaluation results are systematic and explanatory. Therefore, the combination of subjective judgment and objective data is realized, so that the weights of each item are more accurate and reasonable.

## Analytic Hierarchy Process

The calculation process of the AHP is as follows:

Step 1: The judgment matrix is constructed through the pairwise comparison between the factors of each layer. The judgment matrix is determined by an expert group in relevant fields according to the scale method of levels 1~9, as shown in Supplementary Table 1.

**Supplementary Table 1.** Scale 1~9 method of judgment matrix.

| Scale | Meaning |
| --- | --- |
| 1 | Two factors are equally important |
| 3 | Comparing two factors, the former is slightly more important than the latter |
| 5 | Comparing two factors, the former is more important than the latter |
| 7 | Comparing two factors, the former is much more important than the latter |
| 9 | Comparing two factors, the former is extremely important than the latter |
| 2,4,6,8 | The scale values corresponding to the intermediate states between the above two judgments |

The comparison between factor $i$ and $j$ is $x_{ij}$, and the comparison between factor $j$ and $i$ is $x_{ji}=\frac{1}{x_{ij}}$.

The expert group consisted of ten experts, including five women and five men (three professors, five associate professors, and two lecturers). All the experts have long been engaged in farm animal welfare research with rich teaching experience and knowledge. After contacting the experts and obtaining their consent to participate, information was collected by filling in the questionnaire. A nine-point Likert scale was used ranging from 1 = ‘lowest importance’ to 9 = ‘highest importance’.

The judgment matrix $X$ is formally expressed as follows:

$$X=x_{mm}\left[ \begin{matrix} \begin{matrix} x_{11} & x_{12} \\ x_{21} & x_{22} \end{matrix} & \begin{matrix} \cdots& x_{1m} \\ \cdots& x_{2m} \end{matrix} \\ \begin{matrix} \vdots& \vdots\\ x_{m1} & x_{m2} \end{matrix} & \begin{matrix} \ddots& \vdots\\ \cdots& x_{mm} \end{matrix} \end{matrix} \right]$$

Step 2: Normalized to get a vector $Z_{j}$, and the calculation formula is as follows:

$$Z_{j}=\sqrt[m]{x_{j1}x_{j2}\ldots x_{jm}},(j=1,2,\cdots,m)$$

Step 3: Calculate the weight coefficient (and therefore the subjective weight) $S_{j}$ of each evaluation indicator according to the following formula:

$$S_{j}=\frac{Z_{j}}{\sum_{j=1}^{m} Z_{j}},(j=1,2,\cdots,m)$$

Step 4: Consistency index ($CI$) and consistency ratio ($CR$) are used to examine the consistency. The calculation formula of $CI$ is as follows:

$$CI=\frac{\lambda_{max}-n}{n-1}$$

where $\lambda_{max}$ is the largest Eigen root of the judgement matrix, and the calculation formula is as follows:

$$\lambda_{max}=\sum_{i=1}^{m} \frac{{(Xw)}_{i}}{nw_{i}}$$

The larger the $CI$ value, the worse the degree of consistency. When CI = 0, the judgment matrix has completion consistency.

The calculation formula of $CI$ is as follows:

$$CR=\frac{CI}{RI}$$

where $RI$ is the average random consistency index of the matrix, and the $RI$ values of the 1~8 order judgement matrix are shown in Supplementary Table 2. If $CR$ ≤ 0.1, the judgment matrix could be considered to have satisfactory consistency.

**Supplementary Table 2.** Average random consistency index.

| $n$ | 1 | 2 | 3 | 4 | 5 | 6 | 7 | 8 |
| --- | --- | --- | --- | --- | --- | --- | --- | --- |
| $RI$ | 0 | 0.36 | 0.52 | 0.89 | 1.12 | 1.26 | 1.36 | 1.41 |

## Entropy method

The specific application steps of the entropy method are as follows:

Step 1: Assuming that there are $n$ objects to be evaluated and $m$ evaluation indicator, the original matrix can be constructed:

$$X=\left[ \begin{matrix} \begin{matrix} x_{11} & x_{12} \\ x_{21} & x_{22} \end{matrix} & \begin{matrix} \cdots& x_{1m} \\ \cdots& x_{2m} \end{matrix} \\ \begin{matrix} \vdots& \vdots\\ x_{n1} & x_{n2} \end{matrix} & \begin{matrix} \ddots& \vdots\\ \cdots& x_{nm} \end{matrix} \end{matrix} \right],(i=1,2,\cdots,n,j=1,2,\cdots,m)$$

where $x_{ij}$ = the data of the $i$-th objects to be evaluated under the $j$-th evaluation indicator in the original data.

Step 2: In order to avoid the impact of evaluation indicator differences, the original data set is standardized by using the formula:

$$Y_{ij}=\frac{x_{ij}-\min\left( x_{ij} \right)}{\max\left( x_{ij} \right)-\min\left( x_{ij} \right)},(i=1,2,\cdots,n,j=1,2,\cdots,m)$$

where $Y_{ij}$=the data of the $i$-th objects to be evaluated under the $j$-th evaluation indicator after normalization, $\min\left( x_{ij} \right)$=the minimum value in the original data, $\max\left( x_{ij} \right)$=the maximum value in the original data.

Step 3: Calculate the entropy value $E_{j}$ of the $j$-th evaluation indicator using the following formula:

$$E_{j}=-\frac{1}{ln(m)}\sum_{i=1}^{n} p_{ij}\ln\left( p_{ij} \right),(i=1,2,\cdots,n,j=1,2,\cdots,m)$$

where $\ln$ is the natural log, $p_{ij}$ refers to the proportion of the $i$-th objects to be evaluated in the $j$-th evaluation indicator, and the calculation formula is:

$$p_{ij}=-\frac{Y_{ij}}{\sum_{i=1}^{n} Y_{ij}},(i=1,2,\cdots,n,j=1,2,\cdots,m)$$

Step 4: Calculate the entropy weight (and therefore the objective weight) $O_{j}$ of each evaluation indicator according to the formula as follows:

$$O_{j}=\frac{D_{j}}{\sum_{j=1}^{m} D_{j}}=\frac{1-E_{j}}{m-\sum_{j=1}^{m} E_{j}},(j=1,2,\cdots,m)$$

## Comprehensive Weight

The AHP and the entropy method are combined to calculated the comprehensive weight $C_{j}$, and the formula for the calculation is as follows:

$$C_{j}=\frac{S_{j}O_{j}}{\sum_{j=1}^{m} S_{j}O_{j}},(j=1,2,\cdots,m)$$

# Results of weighting

## Subjective weight

The subjective weight of each item is determined by calculating from criterion layer to index layer using the AHP, and the calculation results are shown in Supplementary Table 3. $CR$ of all the items are smaller than 0.1, indicating that the consistency test was passed and the subjective weight obtained by the AHP is reasonable.

**Supplementary Table 3.** Subjective weight of each item calculated by the AHP.

| Target layer | Criterion layer | Weight | Consistency test | Index layer | Weight | Consistency test | Subjective weight ($S_{j}$) |
| --- | --- | --- | --- | --- | --- | --- | --- |
| Public attitudes toward farm animal welfare | Affective attitude | 0.2360 | $\lambda_{max}$ = 3.039  $CI$ = 0.019  $RI$ = 0.520  $CR$ = 0.037 | AFF1 | 0.2013 | $\lambda_{max}$ = 4.183  $CI$ = 0.061  $RI$ = 0.890  $CR$ = 0.068 | 0.0475 |
|  |  |  |  | AFF2 | 0.1708 |  | 0.0403 |
|  |  |  |  | AFF3 | 0.3106 |  | 0.0733 |
|  |  |  |  | AFF4 | 0.3174 |  | 0.0749 |
|  | Cognitive attitude | 0.2703 |  | COG1 | 0.0947 | $\lambda_{max}$ = 6.505  $CI$ = 0.101  $RI$ = 1.260  $CR$ = 0.080 | 0.0256 |
|  |  |  |  | COG2 | 0.1347 |  | 0.0364 |
|  |  |  |  | COG3 | 0.1428 |  | 0.0386 |
|  |  |  |  | COG4 | 0.1421 |  | 0.0384 |
|  |  |  |  | COG5 | 0.2408 |  | 0.0651 |
|  |  |  |  | COG6 | 0.2449 |  | 0.0662 |
|  | Behavioral attitude | 0.4937 |  | BEH1 | 0.1359 | $\lambda_{max}$ = 8.523  $CI$ = 0.075  $RI$ = 1.410  $CR$ = 0.053 | 0.0671 |
|  |  |  |  | BEH2 | 0.1240 |  | 0.0612 |
|  |  |  |  | BEH3 | 0.1072 |  | 0.0529 |
|  |  |  |  | BEH4 | 0.1262 |  | 0.0623 |
|  |  |  |  | BEH5 | 0.1390 |  | 0.0686 |
|  |  |  |  | BEH6 | 0.1327 |  | 0.0655 |
|  |  |  |  | BEH7 | 0.1057 |  | 0.0522 |
|  |  |  |  | BEH8 | 0.1294 |  | 0.0639 |

## Objective weight

The objective weight of each item is calculated using the entropy method, and the calculation results are shown in Supplementary Table 4.

**Supplementary Table 4.** Objective weight of each item calculated by the entropy method**.**

| Item code | $E_{j}$ | $D_{j}$ | $O_{j}$ |
| --- | --- | --- | --- |
| AFF1 | 0.9401 | 0.0599 | 0.0268 |
| AFF2 | 0.9439 | 0.0561 | 0.0251 |
| AFF3 | 0.9300 | 0.0700 | 0.0313 |
| AFF4 | 0.9457 | 0.0543 | 0.0243 |
| COG1 | 0.9370 | 0.0630 | 0.0282 |
| COG2 | 0.8237 | 0.1763 | 0.0789 |
| COG3 | 0.8858 | 0.1142 | 0.0511 |
| COG4 | 0.9186 | 0.0814 | 0.0364 |
| COG5 | 0.8722 | 0.1278 | 0.0572 |
| COG6 | 0.9347 | 0.0653 | 0.0292 |
| BEH1 | 0.9497 | 0.0503 | 0.0225 |
| BEH2 | 0.8780 | 0.1220 | 0.0546 |
| BEH3 | 0.8616 | 0.1384 | 0.0619 |
| BEH4 | 0.8601 | 0.1399 | 0.0626 |
| BEH5 | 0.7188 | 0.2812 | 0.1258 |
| BEH6 | 0.8154 | 0.1846 | 0.0826 |
| BEH7 | 0.7673 | 0.2327 | 0.1041 |
| BEH8 | 0.7823 | 0.2177 | 0.0974 |
